# Chronic kidney disease risk prediction scores assessment and development in Mexican adult population

**DOI:** 10.3389/fmed.2022.903090

**Published:** 2022-10-20

**Authors:** Victor A. Colli, Alejandra González-Rocha, David Canales, Cesar Hernández-Alcáraz, Andrea Pedroza, Manuel Pérez-Chan, Simón Barquera, Edgar Denova-Gutierrez

**Affiliations:** ^1^División Académica de Ciencias de la Salud, Universidad Juárez Autónoma de Tabasco, Villahermosa, Mexico; ^2^Centro de Investigación en Nutrición y Salud, Instituto Nacional de Salud Pública, Cuernavaca, Mexico; ^3^Centro de Investigación en Evaluación y Encuestas, Instituto Nacional de Salud Pública, Cuernavaca, Mexico

**Keywords:** risk score, chronic kidney disease, Mexican, prediction, validation

## Abstract

**Background:**

Chronic kidney disease (CKD) is a major public health problem, with considerable growth in prevalence and mortality in recent years. Screening of CKD at primary care is crucial for the implementation of prevention strategies. The aims of this study are to assess CKD risk prediction scores and to develop a risk prediction score for the Mexican adult population.

**Methods:**

Data from the Mexican National Health and Nutrition Survey 2016 was utilized and 3463 participants ≥ 20 years old were included. Reduced renal function with Glomerular filtration rate and/or the presence of albuminuria was defined as CKD. Multiple logistic regression models were performed for the creation of a training and validation model. Additionally, several models were validated in our Mexican population.

**Results:**

The developed training model included sex, age, body mass index, fast plasma glucose, systolic blood pressure, and triglycerides, as did the validation model. The area under the curve (AUC) was 0.78 (95% CI: 0.72, 0.79) for training model, and 0.76 (95% CI: 0.71, 0.80) in validation model for Mexican adult population. Age, female gender, presence of diabetes and hypertension, elevated systolic and diastolic blood pressure, serum and urinary creatinine, and higher HbA1c were significantly associated with the prevalent chronic kidney disease. Previous CKD risk predictive models were evaluated with a representative sample of the Mexican adult population, their AUC was between 0.61 and 0.78.

**Conclusion:**

The designed CKD risk predictive model satisfactorily predicts using simple and common variables in primary medical care. This model could have multiple benefits; such as, the identification of the population at risk, and prevention of CKD.

## Background

Chronic kidney disease (CKD) is a major public health problem associated with major adverse health events (e.g., greater cognitive impairment, higher prevalence of anemia, hypertension, and metabolic bone disease), progression to kidney failure, and death (1, 2).

Worldwide, in 2017, the prevalence of CKD was 9.1% (95% uncertainty interval [UI] 8.5–9.8), which was roughly 700 million cases. There were 7.3 million (95% UI 5.4–9.2) years of healthy life lost due to disability (YLDs), 28.5 million (95% IU 27.6–29.3) years of life lost (YLLs) and 35.8 million (95% IU 33.7–38.0) disability-adjusted life years (DALYs). There were 1.2 million (95% IU 1.2–1.3) deaths as a result of CKD. In addition, 1.4 million (95% UI 1.2–1.6) deaths from cardiovascular disease (CVD) were attributable to impaired kidney function (7.6% of deaths from CVD) (1, 3). For Mexico, in 2017, the prevalence of CKD was 12.2% (14.5 million cases) (1), 210.9 thousand YLDs, 1.5 million YLLs, 1.7 million DALYs, and 51.4 deaths per 100,000 inhabitants (65 thousand deaths); thus, made this disease the second leading cause of death in that year. From 1990 to 2017, CKD mortality rate increased by 102.3% (2).

Excessive growth in prevalence has been related to accelerated demographic and epidemiological changes (4), such as the high prevalence of Type 2 diabetes (T2D) (15.7%) (5), hypertension (49.4%) (6), overweight, and obesity (75.2%) (7), which are the main risk factors for developing non-communicable diseases and also contribute to deaths from CKD, accounting for half of these deaths.

Up to 98% of people with CKD due to T2D in Mexico are in stages one to three where the disease process can be delayed and controlled, while 2% will require complex and expensive treatments such as peritoneal dialysis, hemodialysis and/or kidney transplantation as replacement and restitutive therapies to survive (stages 4 and 5, considered irreversible) (8). In Mexico CKD is having a significant impact on the finances of the institutions and family economy. In 2014, the average annual health expenditure per person for this disease was US$ 8,966 in the Ministry of Health, and US$ 9,091 in the Mexican Institute of Social Security (9).

Therefore, the first level of medical care is crucial for the implementation of primary prevention strategies aimed at the early and timely control of cardiovascular risk factors, strategies for a stricter control of glycemia and blood pressure, promotion of healthy eating habits, health education, rationalization of the use of potentially nephrotoxic drugs and preventive treatment of hyperfiltration (10). One of the strategies for the timely CKD diagnosis is CKD risk predictive scores, these should be simple but precise and the variables included should be easily accessible in routine clinical settings (11).

The use of risk scores holds promise for large-scale CKD risk stratification and would allow the identification of all segments of the population that would benefit from CKD detection. To date, several studies have shown CKD risk predictive scores, and the predictive capacity of the scores ranges between 0.63 and 0.91 area under the receiver operating characteristic curve (AUROC). However, none have been evaluated in the healthy Mexican population.

Thus, the aims are: (1) to assess previous designed CKD risk prediction scores in Mexican adult population, (2) to develop a training and validation risk prediction score based on the Mexican National Health and Nutrition Survey 2016 (ENSANUT-MC 2016, by its acronym in Spanish).

## Materials and methods

### Design and study population

Data from ENSANUT-MC 2016 was used, a cross-sectional, multistage, stratified, and clustered probabilistic sample of the Mexican population, with national, regional, and urban-rural representation. Detailed survey’s design, sample size calculation and methodology were previously described (12, 13).

For the current study, individuals with at least 8 h of fasting at blood sample collection time were included. In addition, complete serum, urinary biomarker data, and complete survey.

### Sociodemographic variables

This variables and risk factors included were based on self-reported information in the applied survey, such as age, sex, education level (Illiterate, elementary school, high school, and bachelor’s degree), socioeconomic level, place of residence (rural or urban), indigenism and region (center, Mexico City, south and north). Trained and standardized personnel applied the interviews.

### Clinical variables

The trained personnel performed two blood pressure measurements with 30 s difference between each one, with 5 min of rest before the first measurement with the patient seated, an automatic device (Omron HEM-907 XL) was used and the mean of the two measurements was chosen (14). The mean of the two measurements was chosen. Hypertension was considered according to the new criteria of the ISH 2020: systolic blood pressure (SBP) was 140 mmHg or higher and/or diastolic blood pressure (DBP) was 90 mmHg or higher, or when hypertension was self-reported (15). History of kidney stone was measured by self-reporting kidney stone.

### Biochemical variables

Prediabetes was considered when fasting serum glucose was between 100 mg/dl – 125 mg/dl or HbA1c between 5.6 and 6.4%, T2D when fasting serum glucose was 126 mg/dl or greater, or HbA1c was 6.5% or greater, or T2D was self-reported, or use of glucose lowering drugs, and low control of T2D when HbA1c was higher than 7% (16).

The homeostasis model assessment-estimated insulin resistance (HOMA-IR) was used to calculate insulin resistance, it was calculated multiplying fasting plasma insulin (FPI) by fasting plasma glucose (FPG), then dividing by the constant 22.5 [HOMA-IR = (FPI × FPG)/22.5]. The cut-off point (17) to define insulin resistance was higher than 3.80.

Urine albumin was measured with the immunoturbidimetric assay. Urine creatinine was measured using the isotope dilution mass spectrometry (IDMS) standardized methodology, and serum creatinine was measured with the Jaffe method (13). Urinary albumin-creatinine ratio (UACR) was computed and reported in milligrams per gram. Albuminuria was considered when the UACR was 30 mg/g or higher.

### Definition of chronic kidney disease

Reduced renal function [estimated glomerular filtration rate (eGFR) less than 60 mL/min/1.73 m^2^] and/or the presence of albuminuria (UACR) was 30 mg/g or higher were used to define CKD (18).

Glomerular filtration rate (GFR) was estimated using Chronic Kidney Disease Epidemiology Collaboration (CKD-EPI) equation (19).

GFR=141×min(S/c⁢rκ,1)×αmax(S/c⁢rκ,1)-1.209×0.993×A⁢g⁢e1.018[iffemale]×1.159[ifblack]

For this equation: S_*cr*_: serum creatinine (mg/dL), κ: 0.7 for females and 0.9 for males, α: –0.329 for females and –0.411 for males, min indicates the minimum of Scr/κ or 1, and max indicates the maximum of Scr/κ or 1.

### Anthropometric variables

Trained personnel measured height, weight, and waist circumference for all participants. The body mass index (BMI) was calculated from the data of height (m^2^) and weight (kg), and the criteria of the World Health Organization (WHO) was used to classify underweight (<18.5), normal weight (18.5–24.9), overweight (25–29.9) and obesity (higher than 30.0). We define abdominal obesity as; 88 cm or higher waist circumference for men and 102 cm or higher for women (20).

### Chronic kidney disease risk predictive scores

After a review of the literature, scores that had AUROC greater than 0.70 were selected, and included variables to be applied in the first level of medical care, shown in Table 3.

**TABLE 1:** Main characteristics of Mexican National Health and Nutrition Survey 2016, according to the presence of chronic kidney disease.

Variable	Total		No CKD		CKD		*P*-value
	Mean	*SD*	Mean	*SD*	Mean	*SD*	
Age (year)	45.8	17.0	44.7	16.7	56.3	16.3	<0.001
**Sex**							
Male, %	35.0		35.5		29.9		0.05
Female, %	65.0		64.5		70.1		
**Education level**							
Illiterate, %	12.9		12.1		20.1		<0.001
Elementary school, %	67.3		67.1		69.1		
High school, %	13.3		14.0		7.0		
Bachelor’s degree, %	6.5		6.8		3.8		
**Socioeconomic level**							
Low, %	38.1		38.3		37.3		0.387
Medium, %	33.3		33.4		30.1		
High, %	28.6		28.3		31.6		
**Region**							
North, %	21.2		20.6		27.4		0.020
Center and Mexico City, %	42.9		43.3		39.8		
South, %	35.9		36.1		32.8		
**Place of residence (urban and rural)**							
Urban, %	45.1		44.5		50.6		0.038
Rural, %	54.9		55.5		49.4		
**Indigenism, %**							
Yes	12.9		13.3		8.9		0.024
**Anthropometric variables**							
Weight, kg	70.1	15.6	70.1	15.4	70. 3	17.7	0.791
Height, cm	156.2	9.3	156.4	9.2	154.5-	9.6	<0.01
Body mass index, kg/m^2^	28.7	6.2	28.7	6.2	29.3	6.1	0.092
Waist circumference, cm	95.4	13.0	95.2	12.9	98.4	14.1	<0.001
**Clinics and biochemistry variables**							
Systolic blood pressure, mmHg	121.4	19.4	119.7	18.6	132.8	25.7	<0.001
Diastolic blood pressure, mmHg	72.8	10.7	72.6	10.5	75.4	12.8	<0.001
Hemoglobin, mg/dL	14.1	1.9	14.1	1.8	13.3	2.1	<0.001
Serum creatinine, mg/dL	0.74	0.63	0.70	0.15	1.12	2.01	<0.001
Serum total cholesterol, mg/dL	188.8	40.1	188.1	38.7	196.4	52.0	<0.001
HDL-cholesterol, mg/dL	39.2	10.9	39.2	10.8	38.9	11.8	0.729
LDL-cholesterol, mg/dL	112.3	32.1	112.2	31.7	113.9	35.9	0.372
Serum triglycerides, mg/dL	196.5	123.5	194.4	121.0	218.6	144.4	0.001
Fasting plasma glucose, mg/dL	108.4	46.4	105.2	41.0	140.8	76.3	<0.001
HbA1c, %	5.8	1.4	5.7	1.3	6.9	2.2	<0.001
Insulin, mcU/ml	11.2	10.3	11.2	10.5	11.5	8.5	0.617
HOMA-IR^a^ > 3.80	3.1	3.3	3.0	3.3	3.9	3.2	<0.001
Urine creatinine, mg/dL	138.6	80.8	110.6	77.9	1.41.4	80.5	<0.001
Albumin-to-creatinine ratio, mg/g	42.6	93.7	4.3	5.3	436.4	258.1	<0.001
eGFR, ml/min/1.73 m^2^	105.8	27.0	107.6	24.7	87.3	39.4	<0.001
**Family history variables**							
Diabetes, %	35.2		35.1		36.3		0.693
Hypertension, %	39.2		39.6		35.7		0.206
Cardiovascular disease, %	14.0		14.3		11.0		0.137

^a^HOMA-IR, homeostasis model assessment-estimated insulin resistance, HOMA-IR equation = (FPI × FPG)/22.5; FPI, fasting plasma insulin; FPG, fasting plasma glucose.

**TABLE 2:** Prevalence of comorbidities stratified by CKD in the study population.

Variable	Total	No CKD	CKD	*P*-value
**BMI (kg/m^2^), %**				
<25.0	25.9	26.0	24.4	0.193
25.0–29.9	39.4	39.7	36.1	
≥30.0	34.7	34.2	39.1	
**Abdominal obesity, %**				
Yes	80.6	80.0	86.4	0.009
**Diabetes (yes), %**				
Yes	17.2	14.6	44.0	<0.001
**Hypertension, %**				
Yes	27.0	24.1	56.6	<0.001
**Cardiovascular disease^b^, %**				
Yes	4.9	4.6	7.8	0.012
**Kidney stone, %**				
Yes	4.1	3.9	6.6	0.025
Hypercholesterolemia, %	35.3	34.8	40.1	0.065
Low HDL levels, %	57.9	57.8	58.6	0.777
High LDL levels, %	63.9	63.9	64.3	0.090
Hypertriglyceridemia, %	56.8	56.4	61.2	0.100
**Insulin resistance, %**				
High (3.8)	24.5	23.2	38.1	0.001
Albuminuria (UACR ≥ 30), %	7.1	0	81.1	<0.001
**Smoker, %**				
Never	53.0	53.37	49.5	0.071
Former smoker	11.4	11.6	9.1	
Current smoker	35.6	35.0	41.3	

^b^Cardiovascular disease: Acute myocardial infarction, angina pectoris, stroke, heart failure, and other heart diseases. BMI, body mass index.

**TABLE 3:** External models validation in Mexican adult population.

Authors (year)	Population	Model name/ type of model	Variables	Outcomes predicted	AUCROC	Sensitivity (%)	Specificity (%)	External validation in Mexican adult population	Sensitivity (%) in Mexican adult population	Specificity (%) in Mexican adult population
Kwon et al. (21)	Korea	Korean model (KM)/ BLRM	Age (year), sex (female), anemia (yes/no), hypertension (yes/no), diabetes (yes/no), CVD (yes/no), and proteinuria (yes/no).	CKD: eGFR < 60 ml/min/1.73 m^2^ – MDRD and CKD-EPI equation	0.87 (0.84–0.89)	89.4 (84.4–93.2)	70.6 (68.9–72.3)	0.750	51.0	81.0
O’Seaghdha et al. (22)	USA	Model 1: clinical model/ BLRM	Age (year), diabetes (yes/no) and hypertension (yes/no).	CKD: eGFR < 60 mL/min/1.73 m^2^ – MDRD and CKD-EPI equation	0.786	NR	NR	0.744	49.0	78.0
		Model 2: clinical model and baseline eGFR/ BLRM	Age (year), diabetes (yes/no), hypertension (yes/no) and baseline eGFR (mL/min/1.73 m^2^)		0.812	NR	NR	0.762	53.0	85.0
		Model 3: Model 2 plus measure of proteinuria (M3) / BLRM	Age (year), diabetes (yes/no), hypertension (yes/no), baseline eGFR (mL/min/1.73 m^2^), quantitative albuminuria (UACR > 30 or dipstick proteinuria +)		0.813	NR	NR	0.770	55.0	89.0
Al-Shamsi et al. (23)	United Arab Emirates	Full model (FM)/ FGRM	Age (year); sex (male); diabetes (yes/no), hypertension (yes/no), dyslipidemia (yes/no), smoking (yes/no), CVD (yes/no), SBP (mmHg), DBP (mmHg); total cholesterol (mmol/L); triglycerides (mmol/L); HbA1c (%), eGFR (mL/min/1.73 m^2^).	CKD: eGFR < 60 mL/min/1.73 m^2^ for ≥3 months – CKD-EPI equation	0.904 (0.853–0.945)	NR	NR	0.782	56.0	90.0
		Stepwise model (SM) / FGRM	eGFR (mL/min/1.73 m^2^), diabetes (yes/no), cholesterol (mmol/L), and HbA1c (%).		0.918 (0.846–0.964)	NR	NR	0.769	53.0	81.0
Lee et al. (24)	Korea	Model 3 (M3)/ CPHRM	Sex (male), BMI (kg/m^2^), education level, fasting glucose (mg/dL), serum albumin (mg/dL), eGFR (mL/min/1.73 m^2^) and proteinuria (yes/no).	CKD: eGFR < 60 mL/min/1.73 m^2^ for at least two consecutive measurements during follow-up – CKD-EPI equation	0.798 (0.784–0.813)	NR	NR	0.773	58.0	91.0
		Model 4 (M4)/ CPHRM	Sex (male), BMI (kg/m^2^), education level, income, fasting glucose (mg/dL), serum albumin (mg/dL), eGFR (mL/min/1.73 m^2^), proteinuria (yes/no), and Framingham risk score.		0.813 (0.798–0.827)	NR	NR	0.774	53.0	83.0
Nelson et al. (25)	Multinational	Model/ BLRM	Age (year), sex (female), race/ethnicity, eGFR (mL/min/1.73 m^2^), history of CVD (yes/no), ever smoker (yes/no), hypertension (yes/no), BMI (kg/m^2^), and albuminuria (yes/no).	CKD: eGFR < 60 mL/min/1.73 m^2^ – CKD-EPI equation	0.845 (0.789–0.890)	NR	NR	0.757	51.0	82.0
Wen et al. (26)	China	Validation: Simple clinical model BLRM	Sex (female), Waist circumference (cm), Systolic blood pressure (mmHg), diabetes (yes/no), and education (Illiterate/primary school and above).	Predicting incident CKD: reduced renal function or the presence of albuminuria/Albuminuria (UACR ≥ 30 mg/g) and reduce renal function (eGFR < 60 mL/min/1.73m^2^).	0.717 (0.689–0.744)	70.49 (63.30–77.00)	65.14 (61.90–68.30)	0.631	41.0	72.0
Saranburut et al. (27)	Thailand	Model 1 (Clinical) BLRM	Age (year), sex (male), diabetic mellitus (yes/no), systolic blood pressure (mmHg), waist circumference (cm).	Incident cases with decreased eGFR: subjects with preserved GFR (eGFR ≥ 60) at baseline who subsequently developed decreased GFR (eGFR < 60 mL/min/1.73 m^2^) at the 10 years follow-up.	0.71 (0.68–0.74)	NR	NR	0.614	39.0	68.0
		Model 2 (Clinical + limited laboratory tests) BLRM	Age (year), sex (male), systolic blood pressure (mmHg), diabetic mellitus (yes/no), GFR category (ml/min/1.73 m^2^)		0.75 (0.72–0.78)	NR	NR	0.66	38.0	74.0

AUCROC, area under the receiver operating characteristic curve; CVD, cardiovascular disease; SBP, systolic blood pressure (mmHg); DBP, diastolic blood pressure; BMI, body mass index; NR, not reported; m, male; f, female; Y, year; kg/m^2^, kilogram per square meter; mmol/L, millimole per liter; mg/dL, milligrams per deciliter; BLRM, Binary Logistic Regression Model; FGRM, Fine and Gray regression model; CPHRM, Cox Proportional Hazard Regression Model.

For the creation of the novel prediction score the TRIPOD guidelines were followed (Verification Checklist Supplementary material).

### Statistical analysis

Participant’s characteristics are described in percentages if categorical, or in mean and standard deviation if numerical and are compared between subjects with and without CKD using a *t*-test if numerical or using χ^2;^ test if categorical.

Multivariable logistic regression models were fitted to assess how each risk factor contributes to the probability of developing CKD. The data was split into training and validation on an 80/20 ratio. The training dataset was used to fit the predictors on the outcome and the latter to provide an unbiased evaluation of the final models fitted on the validation dataset.

In the training dataset, we fitted different logistic regression models including CKD as the outcome of interest and combinations of age, sex, fasting plasma glucose, SBP, triglycerides, and BMI as predictors. These variables were chosen because we considered them as the most clinically relevant features in order to predict CKD. We selected the model to build our proposed score using the Akaike Information Criterion (AIC).

For training and internal validation of the risk prediction equations among the testing dataset, the AUROC was used, which measures how well the model differentiates those individuals at higher risk of having an event from those at lower risk, a property known as discrimination. Hosmer–Lemeshow χ^2^ tests were also calculated to compare the predicted number of events with the number of events seen. All analyses were done with Stata for windows version 13.0.

## Results

### Main characteristics of the study population

**FIGURE 1:**
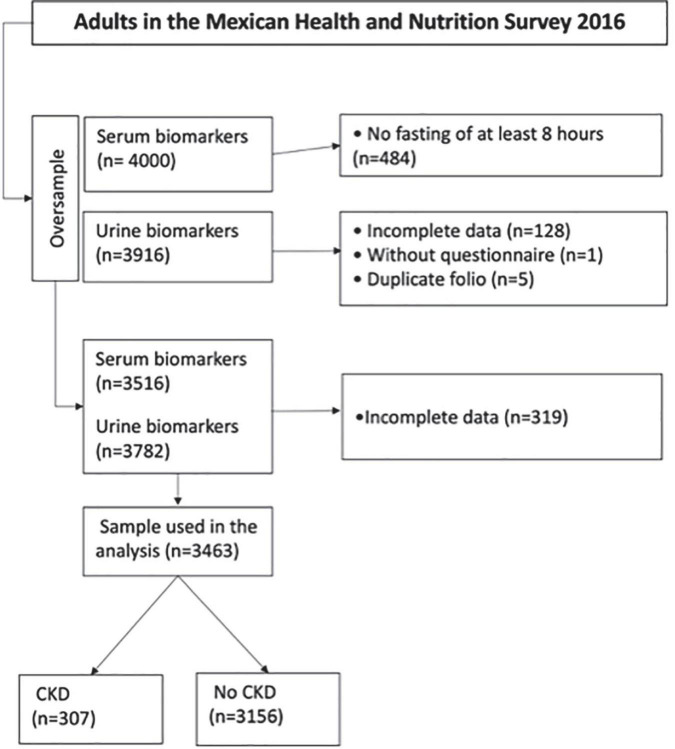
Flowchart of the sample selection.

A total of 3,463 subjects from ENSANUT 2016 MC were included in the final analysis to derive the CKD risk prediction scores (Figure 1). The main baseline characteristics of participants, sociodemographic, clinical, and biochemical, according to the CKD presence or absence, are shown in Table 1. The mean age was 45.8 years, 65.0% were females, 67.3% had elementary school, and 54.9% lived in a rural area. According to anthropometric and clinical measures, those with CKD had a higher mean of BMI compared to those without CKD (29.3 vs. 28.7 kg/m^2^), similar results were observed in the mean of waist circumference (98.4 vs. 95.2 cm), SBP (132.8 vs. 119.7 mmHg), DBP (75.4 vs. 72.6 mmHg), serum creatinine (1.12 vs. 0.70 mg/dL), total serum cholesterol (196.4 vs. 188.1 mg/dL), serum triglycerides (218.6 vs. 194.4 mg/dL), and FPG (140.8 vs. 105.2 mg/dL).

Most of the participants without CKD were overweight (39.7%), while in those with CKD, obesity (39.1%) and abdominal obesity (86.4%) predominated. 14.6% of the participants without CKD lived with T2D, 24.1% lived with hypertension and 23.2% had insulin resistance, while in those with CKD the prevalence was 44.0, 56.6, and 38.1%, respectively (Table 2).

### Assessment of chronic kidney disease risk predictive models

**FIGURE 2:**
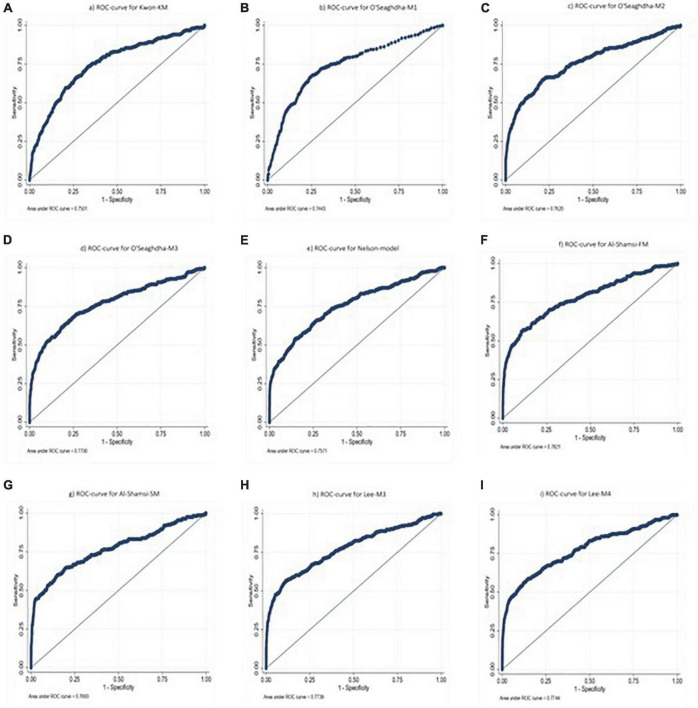
Area under the curve of risk predictive models of chronic kidney disease previously described in the Mexican population, using data of the National Health and Nutrition Survey 2016: **(A)** Kwon Korean model, **(B)** O’Seaghdha model 1, **(C)** O’Seaghdha model 2, **(D)** O’Seaghdha model 3, **(E)** Nelson model, **(F)** Al-Shamsi full model, **(G)** Al-Shamsi stepwise model, **(H)** Lee model 3, and **(I)** Lee model 4.

Chronic kidney disease risk predictive models (21–27) were selected to be assessed with a representative sample of the Mexican adult population, characteristics are shown in Table 3 and the AUROC of the models in Figure 2. The external validation of the different predictive models performed in the Mexican adult population, the AUROC of Kwon et al. model (21) was 0.75; while, in the O’Seaghdha et al. clinical models (22) were 0.74, 0.76, and 0.77 for models 1, 2, and 3, respectively. Additionally, the external validation using Al-Shamsi et al. models (23) showed an AUROC of 0.78 for the first model, and 0.76 the second model. Finally, Lee et al. (24) for model 3 included sex, BMI, level of education, FPG, serum albumin, eGFR and proteinuria, with an AUROC of 0.77 and for model 4 the same variables were included plus Framingham risk score, with an AUROC of 0.77.

### Development of a chronic kidney disease risk predictive model

A model to predict the CKD presence in Mexican adult population was computed. In this sense, the training (1) and validation model (2) included age, sex, BMI, FPG, SBP, and triglycerides. The AUROC for the validation model was 0.76 (95% CI: 0.71, 0.80) and for the training model was 0.78 (95% CI: 0.72, 0.79) (Table 4).

**TABLE 4:** Training and validation risk scores for the development of CKD^c^.

	Training model	Validation model
	**OR (95% CI)**	**OR (95% CI)**
Female	1.44 (1.09, 1.90)	1.56 (1.16, 2.10)
Age	1.03 (1.01, 1.04)	1.04 (1.01, 1.06)
BMI	1.01 (1.00, 1.03)	1.01 (1.00, 1.03)
FPG (mg/dL)	1.02 (1.01, 1.03)	1.02 (1.01, 1.04)
SBP (mmHg)	2.02 (1.54, 2.82)	2.03 (1.51, 2.80)
Triglycerides (mg/dL)	1.01 (1.01, 1.03)	1.01 (1.00, 1.03)
AUC	0.78 (0.72, 0.82)	0.76 (0.71, 0.80)
Sensitivity	75.0	77.1
Specificity	99.2	91.2
Positive predictive value	88.3	89.5
Negative predictive value	93.1	92.1

CI, confidence interval; BMI, body mass index; FPG, fasting plasma glucose; SBP, systolic blood pressure. ^c^CKD defined as: eGFR < 60 ml/min/1.73 m^2^ and albuminuria > 30 mg/g.

### Chronic kidney disease risk prediction algorithms

Prediction equations for training and validation models for CKD are presented below:

Training model:

$$\begin{array}{c} L_{T\,r\,a\,i\,n\,i\,n\,g}=\left(-7.3+0.3646\,i\,f\,W\,o\,m\,e\,n\right)+0.0295\,x\,l\,n\,\left(A\,g\,e\right)\\ +0.0099\,x\,l\,n\,\left(B\,M\,I\right)+0.0198\,x\,l\,n\,\left(F\,P\,G\right)+0.7030\,x\,l\,n\,\left(S\,P\,B\right)\\ +0.0099\,x\,l\,n\,\left(T\,G\right)*P\,{\left(C\,K\,D\right)}_{T\,r\,a\,i\,n\,i\,n\,g}\\ =1-e\,x\,p\,\left(e\,x\,p\,\left(L_{T\,r\,a\,i\,n\,i\,n\,g}\right)\right) \end{array}$$

Validation model:

$$\begin{array}{c} L_{V\,a\,l\,i\,d\,a\,t\,i\,o\,n}=\left(-7.5+0.4446\,i\,f\,W\,o\,m\,e\,n\right)+0.0392\cdot l\,n\,\left(A\,g\,e\right)\\ +0.0099\cdot l\,n\,\left(B\,M\,I\right)+0.0198\cdot l\,n\,\left(F\,P\,G\right)+0.7080\cdot l\,n\,\left(S\,P\,B\right)\\ +0.0099\cdot l\,n\,\left(T\,G\right)*P\,{\left(C\,K\,D\right)}_{V\,a\,l\,i\,d\,a\,t\,i\,o\,n}\\ =1-e\,x\,p\,\left(e\,x\,p\,\left(L_{V\,a\,l\,i\,d\,a\,t\,i\,o\,n}\right)\right) \end{array}$$

*β values taken from ORs reported on Table 4

## Discussion

A predictive risk model was designed for the Mexican adult population using data from the ENSANUT 2016 and it was observed that sex, age, BMI, FPG, SBP, and triglycerides variables predict the CKD risk with the 0.78 AUROC value.

Kwon et al. (21), Al-Shamsi et al. (23), and Lee et al. (24), as the study showed that CKD patients were older, prevalence was higher in T2D and hypertension, mean was higher in serum creatinine and HbA1c, compared to patients without CKD. Two studies (21, 24) reported that the mean total cholesterol, triglycerides and FPG were higher in those with CKD, as was found in this study’s population. The CKD population in Kwon et al. (21) and this study’s, present a higher mean waist circumference and a lower mean hemoglobin compared to those without CKD. For their part, Lee et al. (24) showed that elevated SBP and DBP was significant in CKD patients, as in this study, but not in Al-Shamsi et al. (23). Only this study evaluated HOMA-IR, the mean was 3.0 in patients without CKD and 3.9 in those with CKD, 38.1% of the patients with CKD had insulin resistance.

External validation of all CKD risk predictive models (21–27) was carried out with a representative sample of the Mexican National Health and Nutrition Survey, all of them were population-based, except Al-Shamsi et al. which was clinic-based. The CKD risk predictive models had a fair to good AUROC in their population; however, when replicating these models in our population, the predictive capacity was diminished.

These authors agree with Echouffo-Tcheugui et al. (28) that strategies for early identification and treatment of people with CKD are needed worldwide, and although the model equations incorporate several risk factors that are independently associated with the occurrence of CKD, these should be easily evaluable in routine clinical settings. In this sense, this study’s model for the Mexican adult population was developed with variables which are easily available at the first medical care level. The validation model included sex, age, BMI, FPG, SBP, and triglycerides.

In agreement with the previous studies (21–27), older age, and presence of T2D and hypertension are the main risk factors for developing CKD in stages 3–5. This study’s model uses variables which has been previously used in other predictive models, i.e., age, sex, T2D, and hypertension.

To our knowledge, the present study is the first to assess CKD risk predictive models in an apparently healthy Mexican adult population. The AUROC for this study’s training model was 0.78 and for the validation model was 0.76, compared to Kwon et al. model (21), the AUROC was 0.87 in its primary population and 0.75 in the external validation. O’Seaghdha et al. (22) developed three models, AUROC was between 0.78 and 0.81 in its population, 0.76 in its external validation, and in our validation, it was between 0.74 and 0.77. The AUROC in Al-shamsi et al. (23) two models was 0.90 (multivariate full model) and 0.92 (multivariate stepwise model). They performed very well in their population, however, in the Mexican adult population AUROC was 0.78 and 0.76, respectively. Stepwise model had a lower AUROC than full model, this may be because the stepwise model includes eGFR, T2D, Cholesterol, and HbA1c, but not age, hypertension, triglycerides, and CVD variables, which does include full model and were more significant in Mexican adult population. Finally, Nelson et al. (25) used the CKD Prognosis Consortium (PC), which includes study cohorts from around the world, collecting more than five million patients, and developed two CKD predictive models, one for patients with T2D and the other without T2D. The median C statistic was 0.84 in the cohort without T2D and in his study’s validation, AUROC was 0.75.

It was observed that CKD risk predictive models are characterized by including the main risk factors for developing CKD (older age, T2D, and hypertension); in addition to renal variables (eGFR, proteinuria, albuminuria) that usually improve predictive capacity, and other variables (dyslipidemia, CVD, BMI, sex, etc.). It was considered that the inclusion or not of this group of variables can increase or decrease the predictive capacity depending on the population because they are not always significant.

The objective of creating CKD risk predictive models is to prevent, applied mainly in populations with risk factors for CKD susceptibility, initiation, or progression. The prevalence of this disease has been increasing in Mexico and Latin America, for this reason the need to evaluate CKD risk predictive models, to be used in Mexican adult population and facilitate surveillance of groups susceptible to risk for developing CKD (8).

There are numerous strengths to this study, including the representative based sample, rigorous and detailed assessment of risk factors including measures of renal function and proteinuria. The parsimonious list of variables in the final model is also a significant strength, enhancing the score’s utility and applicability. Some limitations should also be acknowledged. A very high coefficient of variation in creatinine concentrations was observed because creatinine was measured on a single occasion; however, multiple measurements in cross-sectional studies are not feasible. Furthermore, eGFR was estimated using the CKD-EPI equation, which may underestimate eGFR in both healthy individuals and those with CKD. However, a comparison of definitions of incident CKD in the setting of epidemiological research demonstrates that the present definition is the most sensitive (29), which is desirable in view of the potential application of the risk score for population screening. Finally, the cross-sectional design of the study.

## Conclusion

The aims of this study were to evaluate different CKD risk predictive models in the Mexican adult population and develop our predictive risk model. The models evaluated showed a fair to good predictive capacity, however, adjusted in the Mexican adult population, this predictive capacity was diminished. The study’s model is a reliable tool for predict CKD risk among apparently healthy population. It was observed that the variables sex, age, BMI, FPG, SBP, and triglycerides satisfactorily predict the CKD risk, these variables are simple and common in the primary care attention. So, this model could help physicians to identify population at risk. The implementation of CKD risk predictive models will allow the prevention and control of CKD, applied in populations with risk factors for susceptibility, initiation, or progression of CKD. Communication and awareness of the risk to patients is the first step for prevention, it could motivate them to improve their lifestyle and adhere to prescribed therapies. Prevention by identifying patients at risk could also have an economic benefit in our health care system.

## Data availability statement

The datasets used and/or analyzed during the current study are available from the corresponding author on reasonable request.

## Ethics statement

The ENSANUT-MC 2016 protocols were approved by the research, ethics and biosafety committees of the National Institute of Public Health, strictly adhering to the principles set forth in the Declaration of Helsinki. The voluntary nature of participation was recorded in the informed consent and assent forms. The patients/participants provided their written informed consent to participate in this study.

## Author contributions

VC wrote the manuscript (Introduction, Results, and Discussion). AG-R wrote the manuscript (Materials and methods and Discussion). DC contributed the analysis tools. CH-A conceived and designed the analysis. AP wrote the manuscript (Materials and methods) and contributed the data. MP-C contributed the analysis tools. SB conceived and designed the analysis. ED-G conceived and designed the analysis, contributed the analysis tools, and performed the analysis. All authors contributed to the article and approved the submitted version.

## Abbreviations

AIC: Akaike information criterion
AUROC: area under the receiver operating characteristic curve
BMI: body mass index
CKD: chronic kidney disease
CKD-EPI: chronic kidney disease epidemiology collaboration
CVD: cardiovascular disease
DALYs: disability-adjusted life years
DBP: diastolic blood pressure
eGFR: estimated glomerular filtration rate
ENSANUT: National Health and Nutrition Survey
FPG: fasting plasma glucose
FPI: fasting plasma insulin
HOMA-IR: homeostasis model assessment-estimated insulin resistance
IDMS: isotope dilution mass spectrometry
SBP: systolic blood pressure
T2D: type 2 diabetes
UACR: urinary albumin-creatinine ratio
WHO: World Health Organization
YLDs: years of healthy life lost due to disability
YLLs: years of life lost.
